# Heterologous expression in *Caenorhabditis elegans* as an alternative approach to functional studies in *Schistosoma mansoni*

**DOI:** 10.3389/fgene.2014.00120

**Published:** 2014-05-27

**Authors:** Sandra G. Gava, Larissa L. S. Scholte, Ângela Volpini, Riva de Paula Oliveira, Guilherme Oliveira

**Affiliations:** ^1^Grupo de Genômica e Biologia Computacional, Centro de Pesquisas René Rachou, Instituto Nacional de Ciência e Tecnologia em Doenças Tropicais, Fundação Oswaldo Cruz - FIOCRUZBelo Horizonte, Brazil; ^2^Genetics Department, Instituto de Ciências Biológicas, Universidade Federal de Minas GeraisBelo Horizonte, Brazil; ^3^Núcleo de Pesquisa em Ciências Biológicas, Universidade Federal de Ouro PretoOuro Preto, Brazil; ^4^Departamento de Biodiversidade, Evolução e Meio Ambiente, ICEB, Universidade Federal de Ouro PretoOuro Preto, Brazil

**Keywords:** *Schistosoma mansoni*, heterologous expression, *Caenorhabditis elegans*

## Introduction

The use of heterologous expression systems and expression vectors provide a powerful tool for studying the cellular functions of specific genes in their natural cellular environment or in specialized host organisms (Porro et al., 2005). In this context, the gene of interest is cloned in combination with a suitable promoter in a heterologous host (Gräslund et al., 2008). This approach has special relevance in the study of parasitic diseases, such as Schistosomiasis, in which the functional characterization of the parasite is hampered specially because these organisms are not amenable to genetic manipulation and their entire life cycle cannot be cultured *in vitro* (Alrefaei et al., 2011; Suttiprapa et al., 2012; Liang et al., 2013). Although genetic manipulation it is difficult to be achieved in parasitic helminthes, significant advances have been reported towards development of transgenic schistosomes as gene silencing by RNA interference (RNAi) and transient and stable transfection including transgenesis mediated by genome integration using competent vectors or retrovirus (Beckmann and Grevelding, 2012; Mann et al., 2014). Therefore, protein expression in a heterologous host may help the understanding of physiological processes and identifying potential targets with biomedical and biotechnological applications.

*Schistosoma mansoni* is one of the causative agents of human intestinal schistosomiasis. The availability of the genome sequence and a significant amount of transcriptomic and proteomic information allowed the application of a variety of methodologies for the identification and characterization of molecules involved in different physiological mechanisms such as cell signaling essential for the *S. mansoni*'s parasite biology (Knudsen et al., 2005; Curwen et al., 2006; Cass et al., 2007; Guillou et al., 2007).

Protein kinases (PK) play key roles in signaling pathways and have been proposed as potential targets for the development of new anti-schistosome drugs (Dissous et al., 2007). Approximately 1.9% (252 proteins) of the predicted *S. mansoni* proteome corresponds to PKs. However, less than 15% of the kinases have experimental functional evidence, including the JNK subfamily member (Andrade et al., 2011). In contrast to the number of homologs in other species, Andrade and colleagues (2011) have identified only one protein belonging to the JNK subfamily (Smp_172240) in the *S. mansoni* proteome. Knockdown of SmJNK by RNA interference (RNAi) in schistosomula significantly reduced the total numbers of eggs and adult parasites in infected mice (Andrade, 2012). In addition, worms recovered from infected mice showed underdeveloped tegument and reproductive organs (Andrade, 2012). These results indicate that SmJNK might play a role in parasite transformation and survival in the mammal host. However, this line of approach has one methodological limitation. In schistosomes it is not yet possible to conduct functional complementation experiments. In order to advance our knowledge concerning SmJNK gene function we explored the nematode *Caenorhabditis elegans* as a heterologous platform to investigate whether *S. mansoni* JNK represents a physiologically functional protein.

*C. elegans* is a transparent, free-living, soil nematode with 1 mm in length that has emerged as an important animal model in various fields including neurobiology, developmental biology, and genetics. *C. elegans* was the first genome of a multicellular organism to be fully sequenced (The C. *elegans* Sequencing Consortium, 1998). This model offers several advantages, including well-established techniques for genetic and experimental manipulation. Transformation of *C. elegans* has been used to investigate the function of genes from a range of parasitic nematode species, including *tub-1* and *cpl-1* from *Haemonchus contortus, gst-3* from *Onchocerca volvulus* and *fktf-1b* from *Strongyloids stercoralis* (Grant, 1992; Kwa et al., 1995; Britton et al., 1999; Redmond et al., 2001; Britton and Murray, 2002; Kampkötter et al., 2003; Massey et al., 2006).

The *C. elegans* genome encodes five proteins classified as belonging to the subfamily JNK (*jnk-1*, ZC416.4, T07A9.3, Y51B9A.9, and C49C3.10). Based on evolutionary relationships of the JNK protein, *C. elegans jnk-1* was found to be the orthologue of SmJNK (*data not shown*). In *C. elegans, jnk-1* is involved in the modulation of coordinated locomotion (Villanueva et al., 2001). *C. elegans jnk-1* deletion mutants are short-lived, more susceptible to heavy metal and heat stress (Villanueva et al., 2001; Wolf et al., 2008). Overexpression of *jnk-1* increases the resistance to oxidative stress and prolongs the worm's lifespan (Villanueva et al., 2001; Oh et al., 2005). Based on this information, we tested whether the overexpression of SmJNK in *C. elegans* would also result in similar phenotypes, enabling the demonstration that the schistosome enzyme is active and capable of complementing the function of the original gene.

## Strain construction

The expression vector must contain all the DNA sequences necessary for its own expression. The *C. elegans* transcription machinery should be able to recognize and correctly interpret the signals present in these sequences. In order to obtain specific transgenic lineages overexpressing SmJNK, we first constructed the expression cassettes containing the cDNA of *S. mansoni* SmJNK (*Sm_JNK*) downstream of the *C. elegans jnk-1* gene promoter (*Ce_jnk-1p*).

We have chosen to use the *C. elegans* promoter because we had no knowledge if the *C. elegans* transcription machinery would recognize the *S. mansoni* promoter. Additionally, it is possible that gene expression patterns may be differente in two species and the use of the *S. mansoni* promoter may produce a different phenotype due to expression in different cell types or at different levels (Cook et al., 2006). The systematic study of schistosome gene promoters is an area not much explored, but of central relevance for transgenese studies in the field. In *C. elegans*, the majority of protein coding genes are within gene-dense regions of the genome, with cis-acting regulatory regions usually close to the coding region. Consequently, the minimal promoter region required for proper expression of most RNA Polymerase II transcripts lies within a couple of kilobases upstream of the start codon (Okkema and Krause, 2005). For this reason, we selected the 3 Kb region upstream of *Ce_jnk-1* gene as a promoter region. We used *S. mansoni* cDNA to amplify the coding regions to avoid any possibility of incorrect splicing, once its intronic regions could not be recognized by the *C. elegans* splicing machinery. As a positive control, we also construct another transgenic line containing *jnk-1* cDNA of *C. elegans* (*Ce_JNK-1)* under control of the same promoter *Ce_jnk-1p*. The 3'-UTR region was not included due to the lack of exclusive restriction sites in this region.

**Figure 1:**
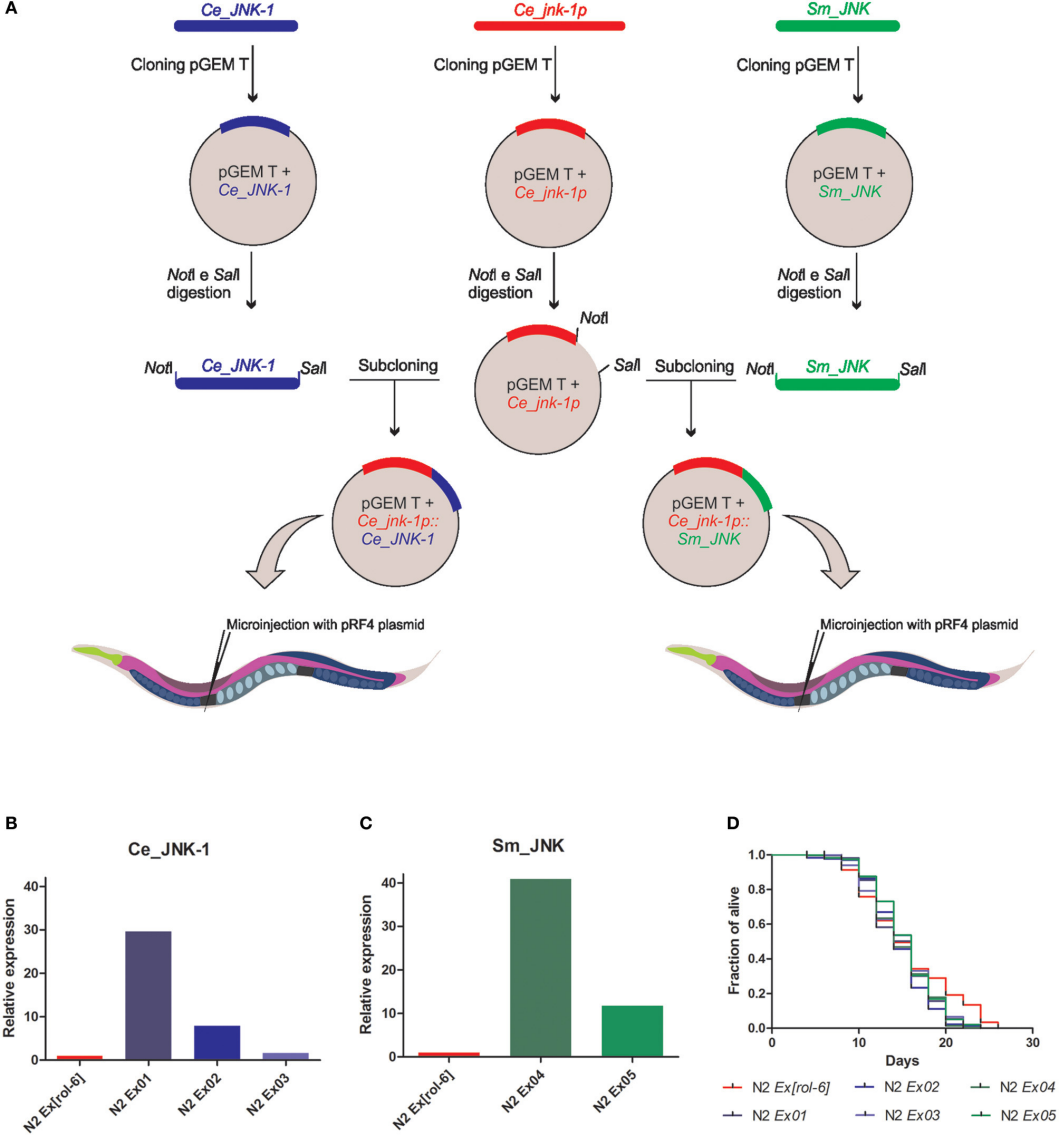
**Experimental cloning strategy and analysis of the expression level and the longevity in *C. elegans* overexpression *Sm_JNK*. (A)** The promoter region of *Ce_jnk-1p* gene was amplified from genomic DNA of adult *C. elegans* N2 Bristol by PCR (5'-GCGCGCAAACTTCCATCTCCTGTTTCTC, 3'-GCGCGCGTGCACAGGATCACACACTTTA). Total RNA was extracted from schistosomula and *C. elegans* adult worms using TRIzol^®^ reagent (Invitrogen) following the standard manufacturer's protocol. CDS of *Sm_JNK* (5'-**GCGGCCGC**ATGGCAAACAACATTCCTCC, 3'-**GTCGAC**TTAATTTTGAATATTACGTA) and *Ce_JNK-1* (5'-**GCGGCCGC**ATGGAGGAACGATTATCCAC, 3'**GTCGAC**TCAGGAATAAATGTCATGGG) were amplified from synthesized cDNA by PCR. Fragments were subsequently cloned in pGEM^®^-T vector. The construction obtained was digested with restriction enzymes (*Not*I and *Sal*I) to linearize the vector containing the promoter region and to recover the CDS. Subsequently, subcloning was performed by ligation of *C. elegans* and *S. mansoni* CDS with the construct containing the promoter region. The plasmids were injected at 50 ng/μL into the gonad of young adult N2 worms to generate stable extrachromosomal transgenic lines. mRNA level of *Ce_JNK-1* **(B)** *Sm_JNK* **(C)** in wild-type animals. mRNA levels were measured in the transgenic lineages obtained for *Ce_JNK-1* (N2 *Ex01[Ce_jnk-1p::Ce_JNK-1*], N2 *Ex02[Ce_jnk-1p::Ce_JNK-1]*, and N2 *Ex03[Ce_jnk-1p::Ce_JNK-1]*) and lineages obtained for *Sm_JNK* (N2 *Ex04[Ce_jnk-1p::Sm_JNK]* and N2 *Ex05[Ce_jnk-1p::Sm_JNK]*). Total RNA of each transgenic lines or N2 worms was isolated from approximately 50 animals using TRIzol^®^ reagent (Invitrogen). cDNA was synthesized using SuperScript™ II Reverse Transcriptase (Invitrogen). RT-qPCR was performed in triplicate with a ABI 7500 RT-PCR system (Applied Biosystems) using SYBR^®^ Green (Applied Biosystems) and the data was analyzed using the comparative Ct method (Livak and Schmittgen, 2001). Relative mRNA levels were normalized to *cdc-42* mRNA levels. **(D)** Worms in L4 stage or young adults were then transferred to new NGM plates containing 0.1 mg/mL 5'-flurodeoxyuridine (FUDR) to prevent progeny growth (Hosono et al., 1982). Animals were tapped every two days and scored as dead when they did not respond to the platinum wire pick. We determined worm's survival from the point when they were transferred to the FUDR plate and lifespan was defined as the account of days that the worms survived starting at day 1. The lifespan assays were repeated three times and statics analysis were done using the Log-rank (Mantel-Cox) test.

The DNA final constructs were delivered to *C. elegans* N2 through intragonadal microinjections (Mello et al., 1991). As previous described by Oh et al. (2005) (Figure 1A), the plasmids were injected at 50 ng/μL into the gonad of young adult N2 worms to generate stable extrachromosomal transgenic lines. Plasmid pRF4 [*rol-6*(*su1066*) plasmid], which has a dominant mutation in *rol-6*, was coinjected at 100 ng/μL for selection by the induction of a dominant “roller” phenotype in the transgenic lineages (Figure 1A). We generated three independent extrachromosomal lineages for *Ce_JNK-1* (N2 *Ex01[Ce_jnk-1p::Ce_JNK-1]*, N2 *Ex02[Ce_jnk-1p::Ce_JNK-1]* and N2 *Ex03[Ce_jnk-1p::Ce_JNK-1]*) and two independent extrachromosomal lineages expressing *Sm_JNK* (N2 *Ex04[Ce_jnk-1p::Sm_JNK]* and N2 *Ex05[Ce_jnk-1p::Sm_JNK]*). We used wild type worms microinjected only with the plasmid pRF4 as negative control. All strains were maintained on nematode growth medium (NGM) plates at 20°C and fed with bacteria of the *E. coli* OP50 strain, as described by Brenner (1974).

## Expression levels and lifespan analysis

Expression of the *Ce_JNK-1* and *Sm_JNK* was detected in all transgenic lines (Figures 1B,C). However, expression levels of the transgene varied substantially among. In the transgenic lines obtained in our study, the expression level of *Ce_JNK-1* ranged from 30 times in the strain *Ce_JNK-1 Ex01* to 1.5 times in the strain N2 *Ce_JNK-1 Ex03* when compared with the control lineage (Figure 1B). The expression level of *Sm_JNK* also varied ranging from 40 times in the strain *Sm_JNK Ex04* to 11 times in the strain *Sm_JNK Ex04* in comparison to the control line (Figure 1C). Any regulatory factor present in *cis* introns were absent in our constructions, once constructions were synthesized from cDNA. It is possible that the addition of such region could further increase the level of expression observed.

Next, we evaluated whether the *Sm_JNK* overexpression could increase the *C. elegans* lifespan (Figure 1D). To monitor longevity under normal conditions, *Ce_JNK-1* and *Sm_JNK* animals were grown at 20°C and scored every two days. Despite the increased expression of *Ce_JNK-1* in transgenic lineages, we did not observe any increase in their longevity as earlier described by Oh et al. (2005). We also did not observe any phenotypic changes in transgenic lines overexpressing *Sm_JNK*. These results could be explained by the absence of introns or 3'-UTR region in the constructs used in this work. As previously described in the literature, the success of phenotype rescue experiments depends on factors such as the presence of regulatory elements in promoter region, the correct processing of *cis*-, and possibly *trans*-splicing, as well as 3' formation of the pre-RNA to produce mature mRNA (Gilleard, 2004). Moreover, the steady-state levels of proteins in eukaryotic cells are also strongly dependent on translational regulatory mechanisms. The overall rate of protein synthesis as well as the translational efficiencies of individual mRNAs are regulated in response to different signals. Therefore, the over expression observed at mRNA level does not may necessarily result in increased protein levels in the correct active form. It is also worth mentioning that the microinjection technique used to obtain transgenic lines carrying repetitive extrachromosomal arrays is relatively fast and efficient. However, one of its drawbacks is that it is difficult to predict and control the level of expression among different arrays resulting in strains with distinct levels of transgene expression. Furthermore, the DNA is injected in the target tissue in an established concentration, but it is not possible to control the amount of DNA successfully injected into each gonad, nor the amount incorporated into arrays in each strain obtained (Evans, 2006).

## Conclusions

Heterologous expression experiments have been performed an alternative approach to characterize schistosome genes. In the present paper, we described for the first time the use of *C. elegans* as an alternative heterologous host to functional studies in *S. mansoni*. The nematode *C. elegans* is more closely related to *S. mansoni* than bacteria, yeast, protozoa, or mammal cells, some of which have been used in heterologous species experiments. The excellent assembly and annotation of the *C. elegans* genome sequence is a valuable resource for studying the developmental and functional biology of parasites.

In addition to the technical difficulties, there are problems regarding to the functional extrapolation of a parasite gene expressed in transgenic *C. elegans*. As all heterologous expression system, one must be careful when extrapolating data, particularly in the functional analysis of distantly related species, which genes conserved in sequence level may be involved in different biological activities (Britton and Murray, 2002). Thus, the ability of a gene from another species to rescue a phenotype in a *C. elegans* mutant does not necessarily imply in a relationship of orthology or does it mean that these genes function in the same way and in similar pathways in both species. Likewise, the failure to recover a phenotype does not necessarily suggest that the genes are involved in different processes. Since there are mechanisms for co-evolution between molecules, genes with similar functions in similar pathways cannot perform its function, because it does not interact with their downstream targets in *C. elegans* (Gilleard, 2004). Nevertheless, the use of *C. elegans* still provides a conceptual and practical framework for functional studies of parasite genes.

An alternative approach to improve the success of *S. mansoni* heterologous expression experiments using *C. elegans* as host would be to perform microinjection using constructs containing: (i) coding regions cloned into vectors specific for expression in *C. elegans*; (ii) synthetically constructed DNA containing the promoter region, the coding region, and 3'-UTR region of the gene of interest.

We hope that the results obtained in this study will contribute to the designing of future experiments that intend to use heterologous expression as an alternative approach to functional studies in *S. mansoni*.

### Conflict of interest statement

The authors declare that the research was conducted in the absence of any commercial or financial relationships that could be construed as a potential conflict of interest.
